# Perception and application of flywheel training by professional soccer practitioners

**DOI:** 10.5114/biolsport.2022.109457

**Published:** 2021-10-25

**Authors:** Kevin L. de Keijzer, Stuart A. McErlain-Naylor, Thomas E. Brownlee, Javier Raya-González, Marco Beato

**Affiliations:** 1 School of Health and Sports Sciences, University of Suffolk, Ipswich, United Kingdom; 2 Institute of Health and Wellbeing, University of Suffolk, Ipswich, United Kingdom; 3 School of Sport, Exercise and Rehabilitation Sciences, College of Life and Environmental Sciences, University of Birmingham, Birmingham, United Kingdom; 4 Faculty of Health Sciences, Universidad Isabel I, Burgos, Spain

**Keywords:** Isoinertial, Strength, Injury, Football, Performance

## Abstract

Growing evidence supports use of eccentric methods for strength development and injury prevention within elite soccer, yet uncertainty remains regarding practitioners’ application of flywheel (isoinertial) methods. The aims of this study were to investigate how the flywheel training literature is perceived and applied by elite soccer practitioners, highlight gaps in knowledge and develop industry-relevant research questions. Fifty-one practitioners completed an electronic questionnaire. Fourteen Likert scale statements were grouped into topics: strength and performance; post-activation performance enhancement and methodological considerations; chronic strength; chronic performance; injury prevention. Three general questions followed, allowing more detail about flywheel training application. A Majority of the participants reported ≥ 2 years’ experience of programming flywheel training. Nearly all participants agreed that familiarisation is needed. Practitioners agree that flywheel training can improve sport performance, strength and likelihood of non-contact injury outcomes. Most practitioners prescribe 2 weekly sessions during pre- and in-season periods. Flywheel sessions mostly consist of squats but a variety of exercises (lunge, hip hinge, and open kinetic chain) are also frequently included. Practitioners are mostly unsure about differences between flywheel and traditional resistance training equipment and outcomes, practicality of flywheel equipment, and evidence-based guidelines. The investigation provides valuable insight into the perspectives and application of flywheel training within elite soccer, highlighting its perceived efficacy for strength and injury prevention.

## INTRODUCTION

Professional soccer match play has shown an increasing frequency of high intensity actions (e.g., sprints, high speed running, accelerations) in recent years, highlighting the need for appropriate training to ensure success [1]. To optimise performance of such actions in matches, practitioners must systematically program resistance training [2], recovery [3], and injury prevention strategies [4]. Resistance training plays an important role for enhancement of strength, performance, and reduction of injury likelihood within professional soccer [5, 6]. However, multiple factors including prolonged national and international travel commitments, fixture congestion, and time dedicated to technical-tactical training often limit the time for strength training [7, 8]. Practitioners have therefore tried to implement different strength training methodologies to efficiently condition athletes. In recent years, flywheel (isoinertial)-based exercise has become more commonly applied by soccer and team sports practitioners as an alternative to traditional resistance training [9, 10].

The flywheel is a resistance training tool that has been employed to enhance strength and performance with success in healthy and athletic populations [11, 12]. The user rotationally accelerates the flywheel during the concentric phase, generating inertial torque that must then be overcome during the eccentric phase [12]. The combination of maximal concentric actions and subsequent high eccentric loads experienced with flywheel training exposes athletes to unique muscular and neural demands [6, 9, 10, 13]. In fact, flywheel training is particularly effective for challenging the eccentric portion of movements, which are often underloaded and difficult to overload with traditional isotonic resistance training methods [6, 9, 14]. Specifically, exposure to intense eccentric training has been shown to enhance motor unit discharge rate and synchronization, as well as selective recruitment of higher-order motor units [13]. The methodological advantages associated with flywheel protocols has increased application as an injury prevention strategy with male soccer players [4, 15, 16]. Moreover, flywheel training has also enhanced acute performance parameters [14, 17–19] within post-activation performance enhancement (PAPE) protocols [20]. Nonetheless, elite practitioners perceive intense eccentric training methods such as the flywheel to be very taxing and difficult to program in-season [6]. In support of this, the current scientific literature does not provide specific considerations for load and risk management when implementing flywheel training in professional soccer [16].

Although flywheel training is applied in a variety of methods in elite team sport environments [9, 12, 20, 21], the perceptions and application of flywheel training methodologies amongst professional soccer practitioners remains unknown. Addressing how flywheel training is applied by practitioners in professional soccer and highlighting their concerns is important to reduce barriers between research and practice [5]. Therefore, the aim of this study was to describe and understand current application and perception of flywheel-based resistance training in professional soccer for acute [20] and chronic adaptations [11, 12] as well as for reduction of non-contact injuries [16].This study is the first to contextualise the way flywheel scientific literature is being applied in professional soccer and to identify whether gaps in current knowledge and application of flywheel training exist. Such an approach has been utilised with a variety of topics associated with elite athlete performance [3, 6]. This study identifies difficulties that practitioners face when applying flywheel training and may be useful for the development of new research questions. Subsequent guidelines may increase practitioners’ confidence in the application of flywheel training [6], further enhancing implementation within professional soccer [4, 6]. We hypothesised that flywheel training exercise prescription and frequency would vary amongst practitioners and would be altered throughout the season.

## MATERIALS AND METHODS

### Participants

Fifty-one practitioners participated in this study, including 21 strength and conditioning (S&C) coaches, 15 sport scientists, 8 fitness coaches, and 7 physiotherapists. Thirty-six worked with male players only, 3 worked with female players only, and 12 worked with males and females. Participants were recruited via the authors’ professional networks and social media platforms. Sample size was maximised through chain sampling, in which participants were encouraged to pass on investigation details to relevant persons within their high-performance soccer networks. The questionnaire was approved by the University of Suffolk (Ipswich, UK) research ethics committee. All participants gave electronic informed consent prior to participation.

### Experimental approach to the problem

Participants completed an electronic questionnaire (hosted online by SurveyMonkey, California, US). A 5-point Likert scale was used for 14 questions, which were grouped into topics and sub-topics: 1) strength and performance, 1.1 PAPE and methodological considerations, 1.2 chronic strength outcomes, 1.3 chronic performance outcomes; 2) injury prevention. The five-point Likert scale (strongly agree, agree, neither agree nor disagree, disagree, strongly disagree) allowed participants to report their level of agreement regarding each statement. Three general application and training questions were also included, allowing practitioners to provide more detail about their application of flywheel training.

### Quantitative Analysis

Frequencies were determined for each Likert-type scale or close-ended question response, with many of the responses also presented as frequency plots. All participants were included in each analysis.

## RESULTS

### Practitioners experience with flywheel devices

Thirty-three participants had ≥ 2 years of experience of programming flywheel training, with a further 14 reporting < 2 years of experience and four having no experience.

### Familiarisation and Post-Activation Performance Enhancement (PAPE)

**FIG. 1:**
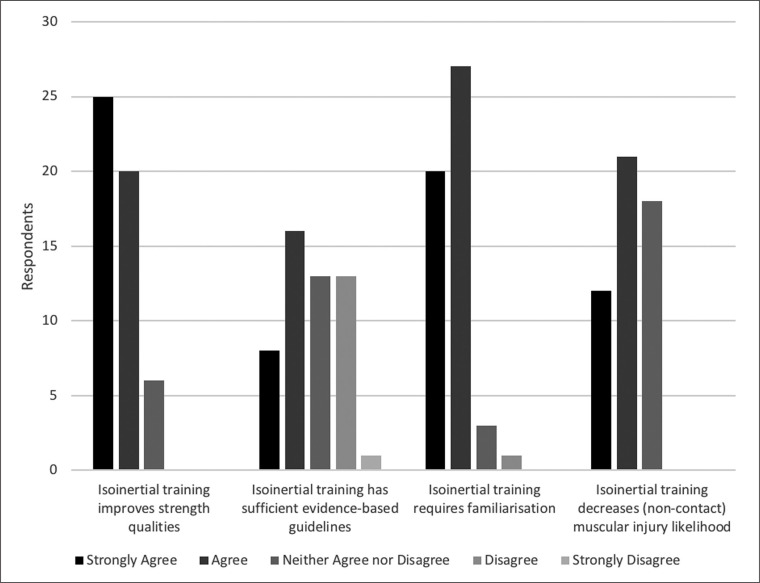
Comparing practitioners’ opinions and perceptions regarding flywheel training evidence based-guidelines, necessity for familiarisation, and for strength and injury prevention (n = 51 for each statement).

**FIG. 2:**
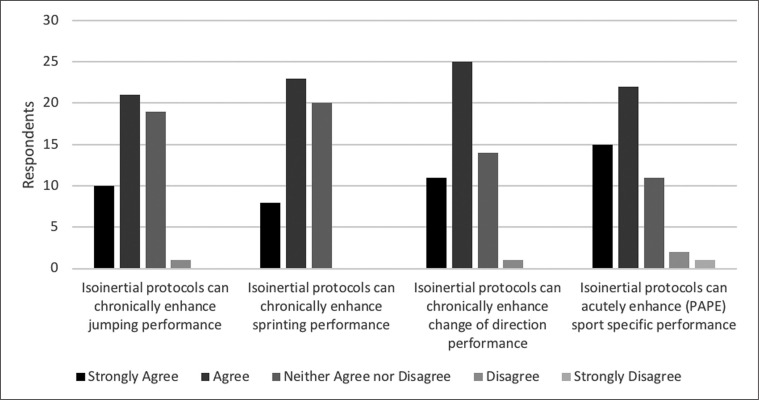
Comparing practitioners’ opinions and perceptions of flywheel training for acute and chronic sport performance enhancement (n = 51 for each statement).

Almost all participants (*n* = 47) agreed familiarisation is necessary to optimise flywheel training, with few neither agreeing nor disagreeing (*n* = 3) and only one single participant disagreeing (Figure 1). One participant did not believe familiarisation sessions are necessary, nine believed one session is needed, 12 participants believed two sessions were necessary, 13 believed three sessions were needed, while nine and two participants stated four and five sessions were necessary, respectively. Finally, five participants also reported that they believe familiarisation is a player dependent process. A majority of participants (*n* = 37) believe that within the scientific literature ‘*flywheel training is well supported for acute sport performance enhancement*’, with some (*n* = 11) unsure and few (*n* = 3) disagreeing (Figure 2).

### Chronic adaptations

**FIG. 3:**
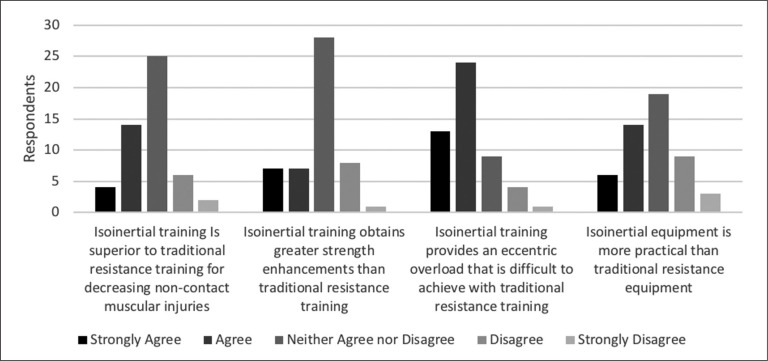
Comparing practitioners’ opinions and perceptions of flywheel training and traditional resistance training (n = 51 for each statement).

Practitioner opinions and perceptions regarding practicality and strength attainment with traditional resistance training and flywheel equipment are reported in Figure 3. More than half of the participants (*n* = 33) agreed that an eccentric overload is necessary during fly-wheel training for acute and chronic adaptations, with some (*n* = 16) remaining unsure, and few (*n* = 2) disagreeing.

**FIG. 4:**
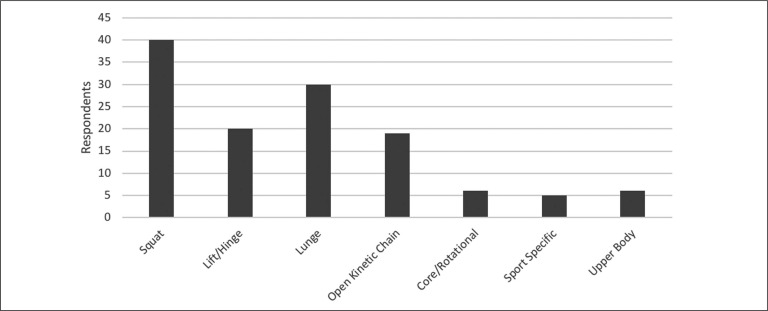
Flywheel exercises that have been programmed by elite soccer practitioners (n = 51).

**FIG. 5:**
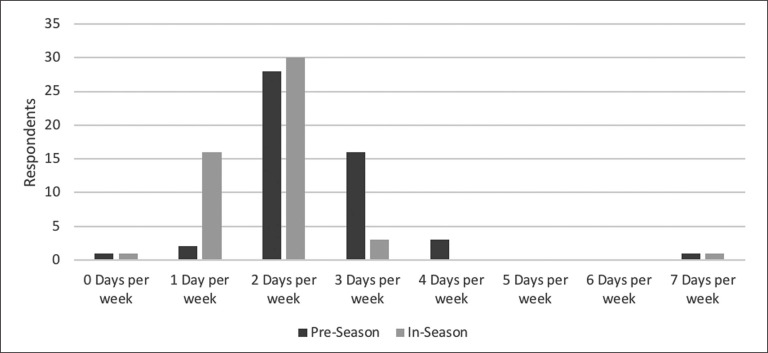
Comparing practitioners’ prescription of flywheel training during the weekly micro-cycle during pre-season and in-season (n = 51 for each statement).

The most frequently programmed flywheel exercise is the squat, with other exercises reported in Figure 4. Practitioners’ views on flywheel familiarisation and effectiveness for increasing strength are reported in Figure 1. Practitioner application did not differ majorly during pre- and in-season periods, is reported in Figure 5.

### Injury prevention

Flywheel training was considered by many (*n* = 33) practitioners to be an effective method of reducing non-contact muscular injuries, with the rest (*n* = 18) remaining unsure (Figure 1). When flywheel training was compared to traditional resistance training methods, some (*n* = 18) believed that flywheel methods were superior while few (*n* = 8) disagreed that flywheel training was superior to traditional resistance training methods (Figure 3). Participants mostly (*n* = 25) stated they neither agreed nor disagreed with the statement.

## DISCUSSION

The aim of this study was to describe and compare the current perception and application of flywheel-based resistance training methodologies in professional soccer for performance and injury reduction purposes. Our findings, which partly agree with our hypothesis, highlight how flywheel training varies in exercise selection (*i.e.,* squat, lunge) and training frequency, among other variables. Practitioners are aware that a familiarisation period is needed to optimize the performance and outcomes with flywheel training. A clear majority of practitioners are confident in the application of flywheel training for acutely and chronically enhancing strength. Although some uncertainty remained, a majority of practitioners believed that flywheel training is useful for decreasing injury likelihood and chronically enhancing change of direction, sprint, and jumping performance. Lacking confidence or awareness of flywheel training guidelines may systematically impact efficacy and application of flywheel training in elite soccer environments. Current perspectives shed light on practical issues and current limitations related to flywheel training for performance enhancement and reduction of non-contact injury likelihood in professional soccer.

### Familiarisation

Although a large portion of practitioners (*n* = 47) agreed that familiarisation is necessary to optimise training, the literature suggests it remains difficult to quantify how many sessions are necessary to achieve reliable outcomes with flywheel devices [9, 22]. Previous studies have reported using either no sessions [23], one [14, 17, 18, 21, 24–26], two [27–30], three [10, 19], or 4–6 sessions [15], and participant dependent familiarisation [31]. A large portion of practitioners (*n* = 25) believe it is necessary to program two or three familiarisation sessions, which is in line with current guidelines [9, 20]. Some practitioners (*n* = 9) believe one familiarisation session is sufficient, possibly due to the limited time for strength training [5] or in reflection of the majority of the literature which employs one session. An equal number of practitioners (*n* = 9) utilise 4 familiarisation sessions. Such sessions may be characterised by lower intensity or volume, as a strategy to mitigate any negative impact of initial flywheel training sessions on concurrent soccer training and performance – although this cannot be confirmed. Few (*n* = 5) practitioners believe familiarisation is dependent on the athleticism, coordination, and training age of the athlete. Although such an approach is sensible, little is published on the topic [9]. Such factors may be particularly important when implementing flywheel methods with youth or novice athletes [32]. Current best practice to enhance familiarisation involves pairing objective data (*i.e.,* velocity outputs) [22], qualitative feedback from the athlete’s movement and athlete confidence in execution.

### Flywheel exercise and PAPE

The majority of practitioners (*n* = 37) believed that PAPE protocols can acutely enhance performance, which is supported by the scientific literature [14, 17, 18]. Desirable neuromuscular responses elicited by flywheel PAPE protocols are related to effective activation of the musculature at a greater velocity and force, improving strength and task specific performance [20]. Nonetheless, limited research on the effects of differing inertial intensities, volume, and exercises on PAPE performance may have impacted practitioners’ beliefs. Some practitioners reported they neither agreed nor disagreed (*n* = 11) and few others stating they disagreed (*n* = 3) that flywheel PAPE protocols acutely enhance sport performance. Nonetheless, comparisons between flywheel PAPE and traditional resistance PAPE squat protocols report similar positive outcomes [14] with comparisons of different inertial loads [9] and movements [18] also attaining similar enhanced outcomes. The aforementioned investigations support practitioner confidence in application of flywheel PAPE protocols to enhance change of direction and jumping outcomes within a variety of contexts [9]. Nonetheless, conclusive evidence on speed performance (≥ 10 m) enhancement within a flywheel PAPE protocol is still needed.

### Chronic application of flywheel training

A large majority of practitioners (*n* = 45) believe that flywheel training is useful for chronically improving strength parameters. Practitioners’ opinions are in agreement with research on flywheel training, which involve several reviews and meta-analyses on various populations [11, 12, 33, 34] and specifically in soccer players [35]. Specifically, the overloaded eccentric phase is perceived to be crucial for most practitioners (*n* = 33) when applying flywheel training. Although some practitioners neither agreed nor disagreed (*n* = 16) and others disagreed (*n* = 2), the perceived importance of a high intensity eccentric contraction can be attributed to the vast evidence supporting its use and well established benefits [9, 12, 34]. Practitioners working within soccer may be particularly attracted to the ability of eccentric training to preferentially recruit high threshold motor units and increase cortical activity – which may boost strength adaptations [13, 25]. In support of current practitioners’ application (Figure 5), weekly and bi-weekly flywheel training has enhanced hamstring strength outcomes with professional and semi-professional soccer players [15, 28, 36]. Although information is still severely lacking on female soccer populations, a recent systematic review highlighted the positive effects of flywheel training on strength related outcomes in females [34].

### Exercise prescription

A high proportion of practitioners (*n* = 40) program squats, which is in agreement with reports of squat-biased eccentric exercise prescription in elite sport [6]. Specifically, few investigations have utilised unilateral [31] and lateral [27, 29, 37, 38] squats, with most prescribing bilateral squats [14, 17–19, 22–25, 29, 30, 36, 39, 40]. Reverse [27] and forward lunges [24, 37], although utilised by many practitioners (*n* = 30), have not been investigated as thoroughly as squats. Nonetheless, bi- and uni-lateral eccentric capacity has been enhanced via flywheel multi-planar movements [27, 29], supporting use of flywheel lunge and multi-directional training (Figure 4). Practitioner utilisation (*n* = 19) of open kinetic chain exercises is supported by effective flywheel leg extension [10] and leg curl [15, 21, 30, 36] protocols in the literature. Even though hamstring based protocols (*e.g.,* leg curl) enhanced performance and injury related outcomes [15, 21, 30, 36], such open-kinetic chain exercises are not as frequently utilised as squats (Figure 4). Training purpose, athlete compliance and experience may all impact exercise selection – although equipment availability is most likely the reason for reduced implementation of open kinetic chain exercises amongst practitioners [5, 31]. Nonetheless, the continued use of evidence based programs involving multiple exercises are recommended for male sporting populations [4, 28, 30, 35].

### Differences between pre- and in-season

The present investigation highlights that a majority of practitioners prescribe flywheel training 2–3 times per week (*n* = 44) and 1–2 times per week (*n* = 46) during the pre- and in-season period, respectively (Figure 5). The reduced training frequency applied from pre- to in-season periods by practitioners is in line with present guidelines [11] and reflects key changes between tactical, technical and physical objectives throughout the soccer season [6, 20]. Apart from athlete, coach, and environmental factors (e.g., team timetables), considerations for exercise choice, intensity, and volume are important for determining optimal training frequency [9, 11, 12]. The application of low volume flywheel protocols [17, 19, 23, 24, 27, 38] may be particularly important during the initial stages of the in-season period if athletes are not accustomed to flywheel training. Careful consideration of training frequency and volume may be important for reducing injury risk [9, 13] and for maintenance of muscle strength and sport performance in-season [38].

### Flywheel training for enhancement of sport specific capacities

Chronic performance enhancement of jumping, sprinting, and change of direction have been achieved with 1–3 weekly training sessions over a 6–10 week period involving 3–6 sets of 6–10 repetitions [15, 24, 26, 36–38]. Practitioners (*n* = 31) mostly agree that jumping, an important capacity in team sports [31], can be enhanced by flywheel training. Although flywheel training has improved jumping performance in highly-trained youth [27, 31, 36–38], semi-professional, and professional male team sport players [23, 24, 26, 28], some practitioners (*n* = 19) stated they neither agreed nor disagreed, while one practitioner disagreed with such statement (Figure 2). Some of the practitioners (*n* = 16) prescribing weekly training sessions during the in-season period may also be encouraged by the literature showing how such exposure can specifically enhance unilateral vertical and horizontal jumping ability after 7–10 weeks of training with youth soccer players [24, 38]. Such a low dose approach may be a viable short-term alternative to precede more comprehensive and time demanding protocols [5] or as a long-term method to maintain vertical jumping performance over a 24 week period with an athletic population at risk of patellar tendinopathies [23].

Most practitioners (*n* = 31) agreed that flywheel training can enhance sprint speed (Figure 2), with evidence supporting such an approach with male youth and professional soccer players and professional handball players [15, 26, 36]. Nonetheless, the rest of the practitioners (*n* = 20) stated they neither agreed nor disagreed, reflecting some inconsistency in the literature [27, 28, 38]. Interestingly, the weekly or bi-weekly exposure utilised in the flywheel soccer literature [15, 27, 28, 36] has also been adopted by many practitioners in the present investigation (Figure 5) – even if such an approach has not always been successful in enhancing performance [27, 28, 38].

A large portion of practitioners (*n* = 36) agree that flywheel training can improve change of direction performance, an important determinant of soccer match play performance [28]. Importantly, practitioner views are in line with evidence supporting flywheel training for enhancement of change of direction performance [15, 27–29, 36, 38]. Eccentric strength, one of several factors associated with successful change of direction performance, can be improved by flywheel training [41]. Investigations lasting 6–11 weeks have enhanced change of direction with semi-professional male soccer players [28], athletes with limited training experience [27], and professional handball players [26]. Nonetheless, some practitioners (*n* = 14) neither agreed nor disagreed and one disagreed that flywheel training can enhance change of direction performance. Considering the evidence supporting the use of flywheel training for enhancing muscle activation and the ability to sustain greater intense deceleration and stabilisation with athletes [27, 30] – it remains unclear why practitioners are lacking confidence in flywheel training for enhancing change of direction performance.

### Comparison between flywheel vs. traditional resistance training

Several practitioners (*n* = 14) believed that flywheel methods were superior to traditional resistance training methods for increasing strength, while the majority (*n* = 28) neither agreed nor disagreed with the statement. Uncertainty amongst practitioners reflects the state of the research [9, 12]. Primarily, a lack of evidence impacts the conclusions drawn [12], with largely contrasting findings also presented [9, 12, 33]. Future high quality study designs (*e.g.,* randomised control trials) are necessary to determine the relative effect of either training modality on strength outcomes. Other comparisons, such as equipment practicality, remain more divided between practitioners – with some (*n* = 20) agreeing, others neither agreeing nor disagreeing (*n* = 19), and fewer practitioners disagreeing (*n* = 12) that isoinertial equipment is more practical than traditional resistance equipment. Although research dedicated to developing application and safety of flywheel training among athletes exists [9], a divide still exists amongst practitioners regarding equipment practicality between the two training modalities (Figure 3). Validated and reliable measures highlighting concentric and eccentric strength during fly-wheel training might not replace traditional strength testing (*e.g.,* isokinetic dynamometry) but may be practically valuable to practitioners due to ease of access [22, 39]. Although quantification of load requires little equipment or time [14, 22], differences between devices and inertial loads may present issues regarding reliability, impacting its applicability [9]. Importantly, flywheel training may also be perceived as a safer and more manageable method than traditional resistance training methods for practitioners working with populations less accustomed or willing to perform intense eccentric training, although opinions may differ between practitioners due to familiarity with flywheel devices [6]. Flywheel devices do not require third-party assistance following an adequate familiarization (*e.g.,* coach) or implements (*e.g.,* chains), enhancing both practicality and safety [6]. In support of this, a majority of practitioners (*n* = 37) believe that flywheel devices provide an eccentric load that is difficult to achieve with traditional resistance training, which is in line with the literature [9]. Although evidence supports such a statement [10, 14], several practitioners neither agreed nor disagreed (*n* = 9) or disagreed (*n* = 5). Differences between devices and techniques may alter eccentric load achieved – possibly swaying practitioners’ opinion on this issue [6, 9, 10].

### Flywheel training and injury prevention

When flywheel training was compared to traditional resistance training for injury prevention, the majority of practitioners (*n* = 25) were not confident that differences existed between the two methodologies. To the best of the authors knowledge, no longitudinal investigation currently exists comparing flywheel training and traditional resistance training for the ability to decrease injury likelihood in athletes [16]. Investigating differences between flywheel and traditional resistance training methods should be performed with elite populations to generate useful evidence for application by practitioners [6]. Nonetheless, a majority of the practitioners (*n* = 33) agreed that flywheel training can help reduce risk and alleviate burden of injuries, with the rest (*n* = 18) neither agreeing nor disagreeing (Figure 1). The importance of consistent intense eccentric training throughout the soccer season is highlighted by the increased risk of muscle damage and injury associated with its prolonged absence (*e.g.,* > 4 weeks) [21]. Although the importance of intense eccentric training is clearly understood by practitioners and researchers alike [4, 13], limited practical evidence exists on practical application of flywheel training with athletic populations [15, 21, 36]. Within soccer, only two such investigations currently exist, with both investigating the efficacy of flywheel training for reducing hamstring injury risk [15, 36]. The investigations prescribed weekly or bi-weekly flywheel squats and/or hamstring curl training protocols [15, 16, 36], which are among the more commonly prescribed exercises by practitioners (Figure 4).

### Guidelines and Application

Nearly half of the practitioners (*n* = 24) stated they were not satisfied with the current guidelines for flywheel training within soccer (Figure 1). Our findings support previous suggestions that a lack of longer duration (*i.e.,* > 12 weeks) protocols and investigations involving elite soccer participants limit practitioner satisfaction with the amount or quality of evidence for males [23]. Flywheel strength training protocols involving female soccer players are also needed to enhance implementation [34]. Specifically investigating training frequency, intensity, exercise choice, and volume may be useful to practitioners – with particular attention also to tracking movement velocity as a means to understand if it can help optimise training outcomes with a variety of movements and devices [22]. Within a PAPE context, future studies investigating the effect of flywheel PAPE protocols on speed performance (≥ 10 m) may enhance practitioner application. Further evidence for enhancement of jumping, change of direction, and sprinting capabilities with elite [41] and female soccer players [20] may also benefit implementation. Since practitioners commonly prescribe training weekly (Figure 5), further investigation into the efficacy of such protocols for sport performance enhancement is also necessary [23, 24, 27]. Such an approach with the objective of enhancing coach/player buy-in and applicability within soccer environments [5] may be a viable short-term alternative or step to progression towards greater weekly application and training outcomes [26] – although this must be thoroughly investigated. Finally, it is possible that some of the practitioners (*n* = 18) who remain unsure about the efficacy of flywheel training for reducing injury likelihood may benefit from seeing further investigation on this topic with elite athletes [16].

#### Limitations and future directions

This study is not without limitations. Although this research may not allow for generalisations to all soccer practitioners due to various types of bias (affecting respondent participation and responses given), it increases awareness of perceived limitations and supports implementation of flywheel training. For example, practitioners, who mostly had ≥ 2 years of experience of programming flywheel training and predominantly worked with males, perceived flywheel methods as effective to generate acute and chronic physical adaptations in soccer environments. Such views are mostly supported by the literature, which boasts several methodological advantages (*e.g.*, combination of repeated maximal concentric and eccentric contractions). Although a clear majority of practitioners agreed on topics such as familiarisation and strength enhancement – mixed responses regarding reduction of injury likelihood, sport performance enhancement, and comparison between methodologies exist. Such uncertainty especially highlights the need for further research into the effects of flywheel training for reduction of injury likelihood and comparison between flywheel and other training methodologies. Furthermore, practitioners believe that evidence-based guidelines are lacking, which may heavily influence the efficacy of flywheel training within soccer. The present investigation does not report different familiarisation nor programming strategies when utilising flywheel training with youth or adult soccer players. Nonetheless, further work dedicated to developing evidence-based recommendations for flywheel training implementation within male and female soccer is needed.

## CONCLUSIONS

Practitioners agree that flywheel training can improve sport performance, strength, and likelihood of non-contact injury outcomes. Most practitioners prescribe 2 weekly sessions during pre- and in-season periods. Flywheel sessions mostly consist of squats, but a variety of exercises (lunge, hip hinge, and open kinetic chain) are also frequently included. Practitioners are mostly unsure about differences between flywheel and traditional resistance training outcomes, practicality of flywheel equipment, and evidence-based guidelines. The investigation provides valuable insight into the perspectives and application of flywheel training within elite soccer, highlighting its perceived efficacy for strength and performance outcomes.

### Practical Applications

Flywheel training is utilised by practitioners for various purposes within soccer environments. Practitioners initially dedicate 2–3 fly-wheel training sessions to familiarisation, especially if the athlete lacks flywheel training experience. The pairing of flywheel devices and technology (e.g., tablets) to permit instantaneous feedback may enhance individualisation and outcomes – especially during familiarisation. Although flywheel and traditional resistance training are both deemed valid for enhancing performance and strength parameters, advantages of one methodology over the other remain unclear. Practitioners typically prescribe 2–3 and 1–2 weekly flywheel sessions during the pre- and in-season period, respectively. Within these sessions, practitioners confidently utilise a variety of exercises for chronically enhancing performance and strength – while also prescribing flywheel PAPE protocols to acutely enhance performance. Although some evidence supports use of flywheel training (i.e., leg curl protocols) to reduce injury risk amongst soccer players, limited use by practitioners highlights potential practical issues related to implementation (e.g., time or equipment available).
